# Effects of *Cirsium japonicum* var. *maackii* on avelliation of metabolic disease by improving insulin resistance

**DOI:** 10.1186/s42826-025-00234-w

**Published:** 2025-01-16

**Authors:** Hye-Bin Yoon, Yuseong Jang, Hyeon-Gi Paik, Hwal Choi, Jihye Choi, Jungkee Kwon

**Affiliations:** Department of Laboratory Animal Medicine, College of Veterinary Medicine, Jeonbuk National University, The 1st Veterinary R&D Building Rm 301, 79 Gobong-ro, Iksan-si, Jeollabuk-do 54596 Republic of Korea

**Keywords:** *Cirsium japonicum* var *maackii*, Metabolic syndrome, Type 2 diabetic, *db/db* mice

## Abstract

**Background:**

Metabolic syndrome (MetS) refers to a group of risk factors that cause health problems, such as obesity, diabetes, dyslipidemia, and hyperglycemia. MetS is characterized by insulin resistance, which leads to abnormal insulin sensitivity. *Cirsium japonicum* var. *maackii* (CJ) is perennial herbaceous species found in Asia that exhibits antioxidant, antidiabetic, antitumor, antifungal, and anti-inflammatory activities. In this study, we aimed to measure the effects of CJ on MetS by improving insulin resistance in a db/db type 2 diabetes mouse model. After administrating CJ extract (CJE) for db/db mouse for 6 weeks, we measured with the evaluation of Insulin resistance, lipid profiles, histological analysis of liver, damage of liver and kideny.

**Results:**

The results showed that CJE was effective in reducing body weight and fat mas and showed a positive effect on lowering blood glucose and improving insulin sensitivity. CJE improved dyslipidemia by increasing serum-HDL levels and decreasing serum-LDL levels. In addition, CJE reduced liver and kidney damage in histological analysis.

**Conclusions:**

These results demonstrate the anti-diabetic effects of CJE and suggest its potential for improving MetS. Therefore, CJE may have potential values as a functional food material for managing MetS.

## Background

Metabolic syndrome (MetS) refers to complex of interrelated risk factors for cardiovascular disease (CVD) and diabetes [1]. These factors include hyperglycemia, increased blood pressure, elevated triglyceride (TG) levels, and low high-density lipoprotein (HDL) cholesterol levels, all of which can lead to obesity. MetS is diagnosed when an individual present with more than three of these four factors. The prevalence of MetS is approximately 25%, with a higher incidence in the older population, and it is increasing annually. Obese males and females are 5.5 and 6 times more likely to have MetS than those who are underweight or normal weight [2]. Many chronic diseases are related to MetS, including type 2 diabetes mellitus (T2DM), CVD, chronic kidney disease (CKD), and non-alcoholic fatty liver disease (NAFLD) [3].

Insulin resistance, a key feature of Mets, plays a significant role in the pathophysiology [4, 5]. It involves a defects in the insulin receptor or signaling pathways, leading to an inability of target tissues to regulate blood glucose levels normally. This includes disruptions in key processes such as inhibiting lipolysis and glycogen synthesis [6]. Insulin resistance also contributes to abnormal mitochondiral function, generating oxidative stress through reactive oxygen species (ROS), which reduces oxidative capacity and results in lipid addmulation [7]. Several factors contribute to insulin resistance, including overeating, obestiy, hyperglycemia, elevated free fatty acid (FFA) levels, and other metabolic disorders [8]. The db/db mouse, a leptin receptor mutant characterized by high plasma triglyceride and cholesterol levels, is widely used as a genetic model for studying MetS, T2DM, and insulin resistance. Due to its phenotypic similarities to human metabolic conditions, it serves as a valuable tool for investigating the mechanisms underlying obesity, insulin resistance [9–11].

For treatment of MetS or T2DM, drugs are available to manage or alleviate patient symptoms; however, such drugs can have side effects. For example, thiazolidinediones can cause hypoglycemia and weight gain [12], and SGLT2 inhibitors are associated with urinary and genital infections and ketoacidosis [13]. For these reasons, there is currently active research into natural substances with mechanisms similar to those of drug formulations that may exhibit activities such as regulating insulin secretion, improving insulin resistance, and enhancing insulin sensitivity, with fewer side effects [14, 15].

CJ is a wild annual herbal plant of the composite family that is found in many areas of Korea, China, and Japan. CJ contains polyphenols that positively affect glucose homeostasis and insulin resistance [16]. CJ has been used for a long time to treat tumors such as uterine cancer, and leukemia and as an antihemorrhagic, antihypertensive, antihepatitic, and diuretic agent [17]. CJ also exhibits many other biological activities, including hepatoprotective and vasoconstrictor properties. Various studies have reported that CJ extract improves lipid metabolism [18], inhibits lipid peroxidation [19], and has antioxidant and antidiabetic activities. Moreover, our previous study showed that CJ inhibits the advanced glycation end products (AGEs)/receptor AGEs (RAGE) pathway in streptozotocin (STZ)-induced type 1 diabetes mellitus rats [20]. However, no study has examined the effects of CJ on MetS and insulin resistance. Therefore, in this study, we investigated the effects of CJ extract (CJE) on MetS, characterized by insulin resistance, in *db/db* mice.

## Methods

### Preparation of CJE

CJ was obtained from Rural Development Administration, Jeonbuk, Korea. The CJ was dried at 60 °C and powdered. A total of 100 g of CJ power was refludxed with water (w/v = 1:20) for 3 days at room temperature. After filtering, the residue was re-extracted, filtered, and evaporated using a rotary evaporator at 45 °C. CJE was stored at 4 °C, and CJE suspension in PBS was freshly prepared daily before treatment.

### Animals and experimental design

C57/BL-KsJ-(db/db) mice (*n* = 24) and C57BL/KsJ-(m+/db) mice (*n* = 6) were purchased from Japan SLC Inc. (Shizuoka, Japan). All mice had free access to food and water during the experimental period. The animal laboratory was maintained at 22 ± 1˚C and 50 ± 10% humidity, with 12 h light and dark cycles. After one week of acclimatization, all experiments were performed on 6-week-old male mice. After the adaptation week, the mice were randomly divided into five groups: m/m (m+/db, normal control group), Veh (db/db, negative control group), glimepiride (GLM 2.5 mg/kg/day, positive control group) [21]. C100 (CJE 100 mg/kg/day), and C200 (CJE 200 mg/kg/day) groups. The samples were dissolved in PBS buffer and administered orally at the same time every day, with the same amount of PBS buffer given to the m/m and Veh group. Each group received the treatment regimen for 6 weeks. All animals were cared for in accordance with the Jeonbuk National University institutional guidelines for the care and use of experimental animals. Animals were weighed at same time each week. All protocols for the animal experiments were approved by the Institutional Animal Care and Use Committee of Jeonbuk National University, South Korea (JBNU 2020 − 0174).

### Measurement of glycemia controlling parameters

Fasting blood glucose levels were measured once a week during the treatment period after 12 h of fasting. Blood glucose levels were measured with Accu-Check (Roche Diagnostics Korea Co, Ltd, Seoul, Korea) following the manufacturer’s instructions. An OGTT was performed in the 5th week of treatment after 12 h of fasting. Blood was sampled from the tail vein of mice at time points 0, 30, 60, and 120 min after an oral glucose load of 1 g/kg of body weight.

An ITT was performed in the last week of treatment after 12 h of fasting. Diluted regular human insulin (Humulin R; Eli Lilly Japan K.K., Hyogo, Japan) was injected intraperitoneally into mice at a dose of 1 unit/kg of body weight. Blood was sampled from the tail vein of mice at baseline (0 min) and 30, 60, and 120 min after the insulin injection. Food was withheld from the cage during the injection period.

### Organ weight and biochemical analysis of serum

At the end of the experiment, all animals were fasted for 12 h. Mice were anesthetized with isoflurane (Hana Pharm Co., Ltd., Seoul, Korea). Blood samples were immediately collected from retro-orbital sinus/plexus sampling, and serum was separated by centrifuging the blood samples at 3,000 g for 15 min. Serum samples were frozen at − 70 °C for further analysis. Liver and fat samples were harvested after euthanasia using isoflurane, and organs were weighed and processed.

Total cholesterol (TC), high-density lipoprotein cholesterol (HDL), low-density lipoprotein cholesterol (LDL) (Cell Biolabs, San Diego, CA, USA), triglycerides (TG, Cayman Chemical, USA), serum insulin (APLCO, Salem, NH, USA), and serum ROS (Cayman Chemical, USA) were measured by ELISA kits using a microplate reader (Bio Tek, USA) following the manufacturer’s instructions. Serum aspartate aminotransferase (AST), alanine aminotransferase (ALT) (Asan Pharm, Hwaseong, Korea), creatinine (Sigma, St. Louis, MO, USA), and blood urea nitrogen (BUN) (Cosmo Bio, Tokyo, Japan) were measured using ELISA kits with a microplate reader (Bio Tek, USA) following the manufacturer’s instructions.

The homeostasis model assessment of insulin resistance (HOMA-IR) is calculated as the product of fasting glucose and fasting insulin divided by a constant, with a value of 1 representing normal insulin sensitivity [22, 23]. The insulin resistance index was calculated using the homeostasis model assessment of insulin resistance (HOMA-IR) as follows:

$$\eqalign{& {\rm{HOMA}} - {\rm{IR}} = \cr & {{({\rm{ fasting insulin in }}mU/L) \times ({\rm{ fasting glucose in mmol }}/L)} \over {22.5}} \cr}$$

### Histological analysis and triglyceride analysis in the liver

Liver tissues were homogenized in 1 mL of PBS. Homogenates were extracted with 5 mL of chloroform/methanol mixture. After vigorous vortexing, the mixture was separated into two phases and centrifuged at 2,500 rpm at 4 °C for 15 min. Total hepatic triglyceride (TG) concentrations were standardized to the respective protein concentrations and are presented as mg of lipid per g of tissue protein. Intracellular TG contents were measured using a commercial TG ELISA kit (Cayman Chemical, Ann Arbor, MI, USA) following the manufacturer’s instructions.

For histopathology analysis, liver tissues were trimmed and fixed in 4% paraformaldehyde (PFA) for 48 h. The fixed samples were embedded in paraffin and cut into 4–5 micron sections. The sections were deparaffinized with xylene, rehydrated with a graded alcohol series, and stained with hematoxylin and eosin (H&E). H&E-stained sections were imaged, and the areas of stained regions were quantified in pixels using the Image Pro analysis program.

### Statistical analysis

All data were obtained from at least three individual experiments. Data are expressed as mean ± standard error (SE). Statistical analysis between groups was performed using one-way analysis of variance (ANOVA) using Prism 5 software (GraphPad Software version 5.0., San Diego, CA, USA). Statistical significance was set at *p* < 0.05.

## Results

### Effect of CJE on body weight and epididymis fat

**Fig. 1:**
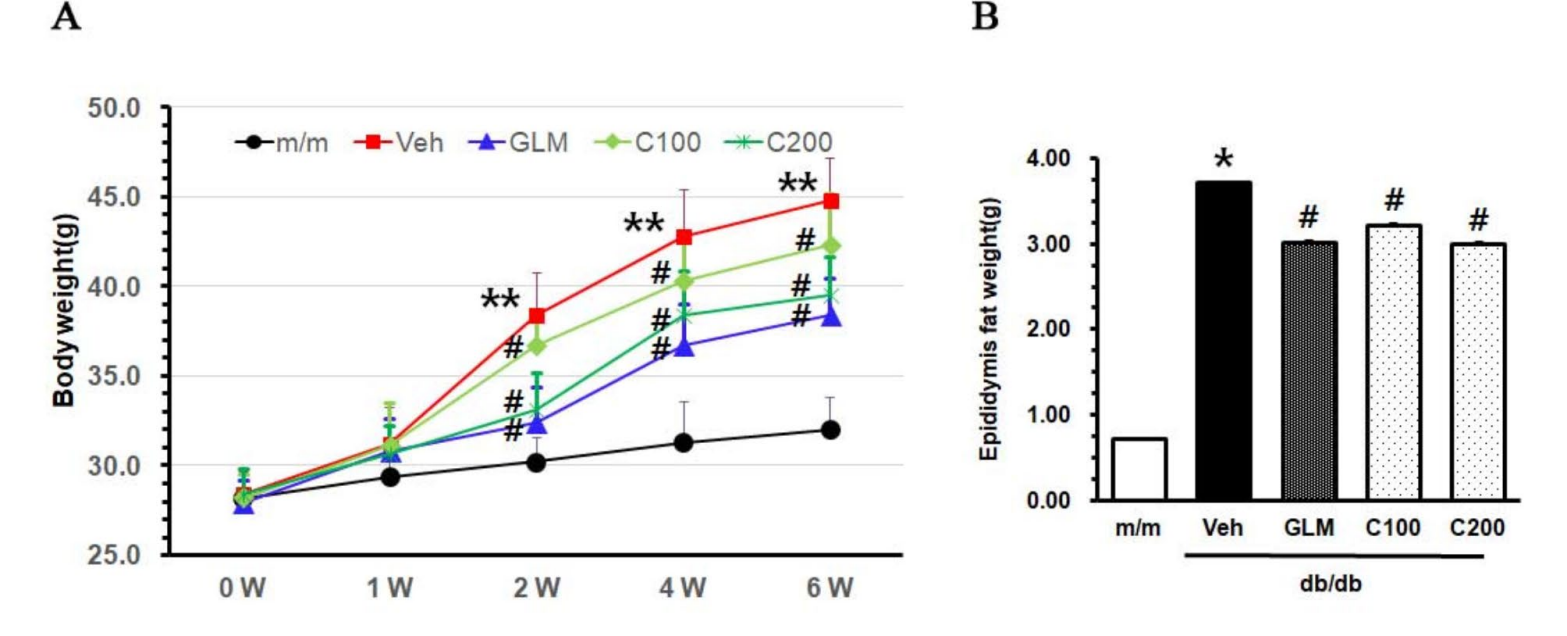
Changes in body and epididymis fat weight. (**A**) The body weight changes for 6 weeks. (**B**) Weight of epididymis fat collected after 6 weeks. Data are expressed as the mean ± SE (*n* = 6, *p < 0.05 vs. m/m, **p < 0.01 vs. m/m, #*p* < 0.05 vs. Veh). m/m: Normal mice; Veh: non-treated db/db mice; GLM: positive control db/db mice; C100: CJE (100 mg/kg/day)–treated db/db mice; C200: CJE (200 mg/kg/day)–treated db/db mice

At the start of the experiments, there were no differences in body weight among the groups of db/db mice. During the 6-week period, the body weight increased steeply in m+/db mice. The body weight of the Veh group was significantly higher compared with that of the m/m group (*p* < 0.01). CJE-treated db/db mice showed a significant reduction in body weight from 2 weeks compared with the Veh group in a dose-dependent manner (Fig. 1A). After 6 weeks of CJE treatment, mice were sacrificed and the weight of epididymis fat was evaluated. The weight of epididymis fat in the CJE-treated group decreased compared with the Veh group in a dose-dependent manner (Fig. 1B). The GLM group effectively reduced body weight and epididymis fat weight, while the CJE 200 group exhibited a comparable trend. These results indicated that CJE countered the increase of body weight and epididymal fat in the diabetic mice model.

### Effects of CJE on insulin resistance

**Fig. 2:**
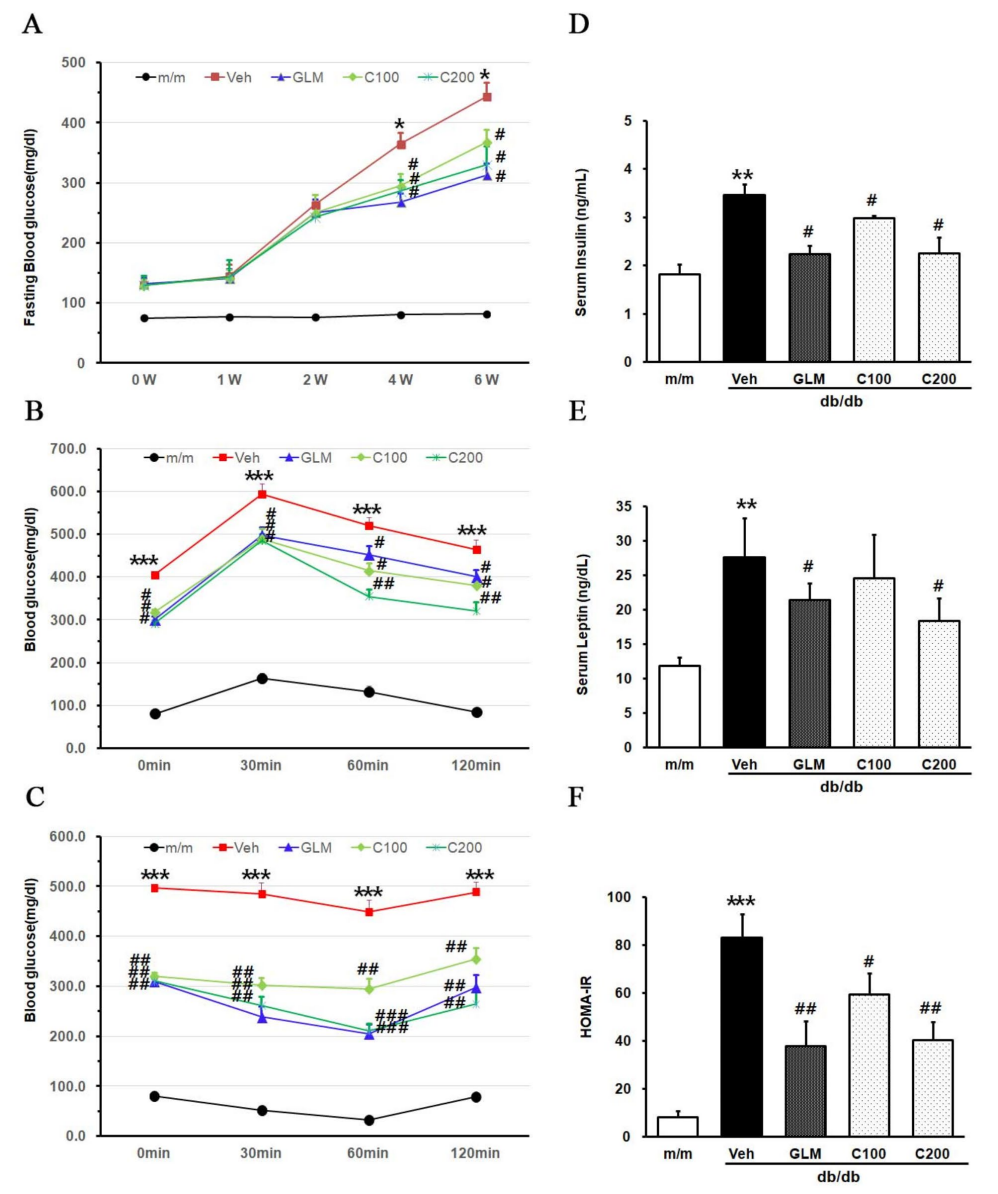
Effect of CJE on insulin resistance in db/db mice. (**A**) Fasting blood glucose. (**B**) Glucose tolerance test. (**C**) Insulin tolerance test. (**D**) Serum insulin levels. (**E**) Serum leptin levels. (**F**) Homeostatic model assessment of insulin resistance (HOMA-IR). After 5 weeks of CJE treatment, OGTT was evaluated. After 6 weeks of CJE treatment, ITT was evaluated. Data are expressed as the mean ± SE (*n* = 6, *p < 0.05 vs. m/m, ***p < 0.001 vs. m/m, #*p* < 0.05 vs. Veh, ##*p* < 0.01 vs. Veh, ###*p* < 0.001 vs. Veh)

We investigated the effects of CJE on insulin resistance in db/db mice. The effect of CJE on fasting blood glucose levels is shown in Fig. 2A. The fasting blood glucose levels of the Veh group significantly increased compared with the m/m group, whereas CJE-treated groups showed a decreased rate of increase from 4 weeks. Glucose tolerance was monitored by an OGTT at 5 weeks after treatment with CJE (Fig. 2B). In the Veh group, OGTT values peaked at 30 min after glucose administration and then decreased. The OGTT levels at 30 min of the other experimental groups were lower than those of the Veh group. In CJE-treated groups, OGTT levels were reduced at 60 and 120 min after glucose administration in a dose-dependent manner. The C200 group showed OGTT levels at 120 min, especially compared with the GLM group. After administration of glucose, the rates of increase in the blood glucose concentration were similar among the groups during the first 30 min. The blood glucose concentration was significantly higher in the Veh group compared with the CJE and GLM groups.

In the 6th weeks of treatment, an insulin tolerance test (ITT) was performed (Fig. 2C). The CJE groups showed a significant reduction in ITT level compared with the Veh group. The effect of CJE treatment on insulin levels in db/db mice is shown in Fig. 2D. The Veh group exhibited higher serum insulin levels compared with the m/m group, whereas the C200 group showed insulin levels similar to those of the GLM group. This suggests that CJE efficient in lowering insulin levels like GLM. The effect of CJE treatment on serum leptin levels is shown in Fig. 2E. The C200 group had significantly lower serum leptin levels compared with Veh group (*p* < 0.05). The HOMA-IR levels in db/db mice were significantly decreased by CJE in a dose-dependent manner (Fig. 2F).

### Effect of CJE on dyslipidemia

**Fig. 3:**
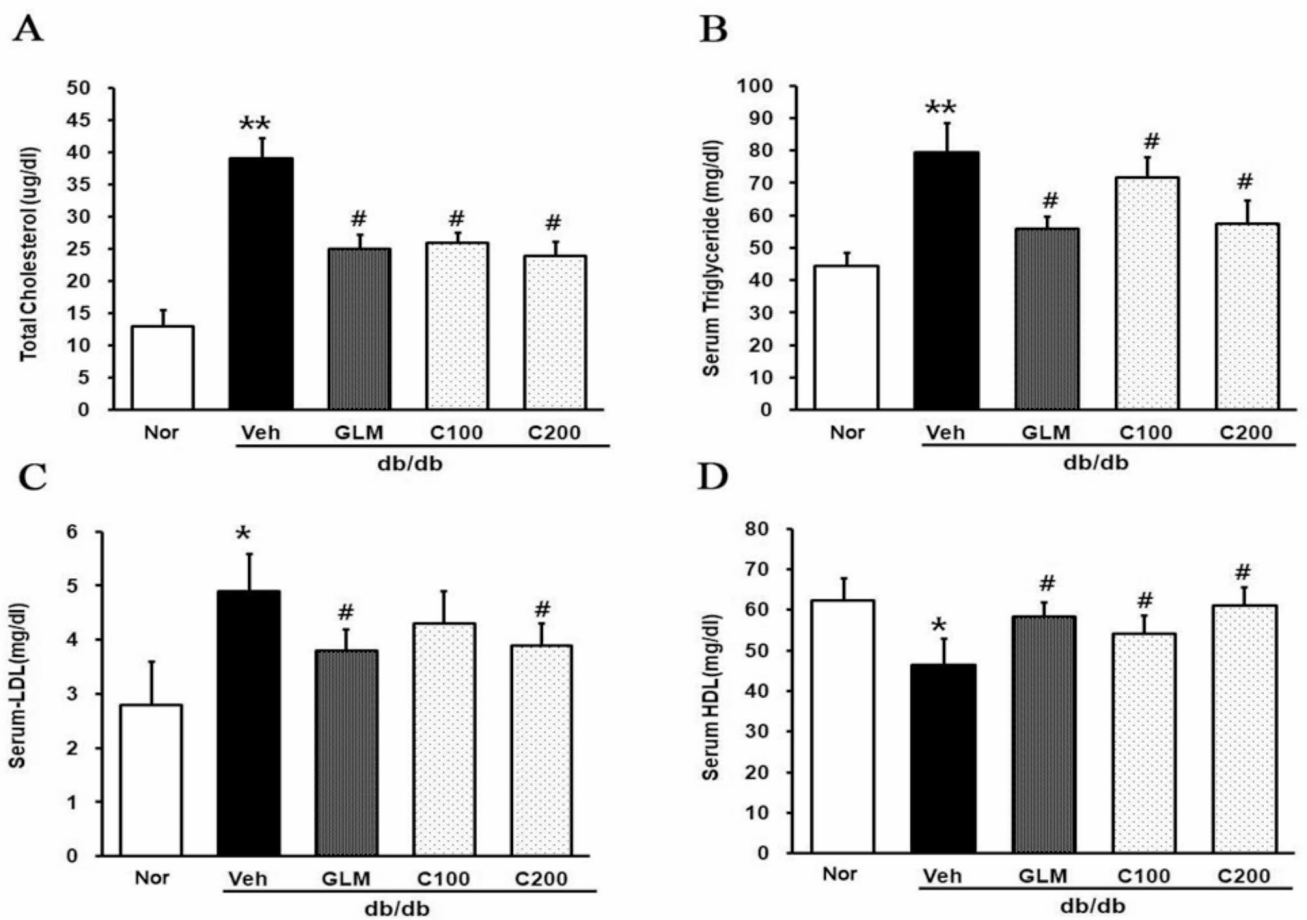
Effect of CJE on lipid profiles in db/db mice. (**A**) Total cholesterol (TC). (**B**) Triglyceride (TG). (**C**) Low-density lipoprotein cholesterol (LDL-C). (**D**) High-density lipoprotein cholesterol (HDL-C). Data are expressed as the mean ± SE (*n* = 6, *p < 0.05 vs. m/m, **p < 0.01 vs. m/m, #*p* < 0.05 vs. Veh)

We examined the effect of CJE on lipid profiles (Fig. 3). The serum concentration of TC, TG, and LDL cholesterol was significantly increased in the Veh group compared with the m/m group. Notably, the serum concentration of TC, TG, and LDL cholesterol was lower in the CJE groups compared with the Veh group in a dose-dependent manner (Fig. 3A and B, and 3C). The CJE-treated mice showed increased HDL cholesterol levels compared with the Veh group (Fig. 3D).

### Effect of CJE on histological changes and lipid accumulation in the liver

**Fig. 4:**
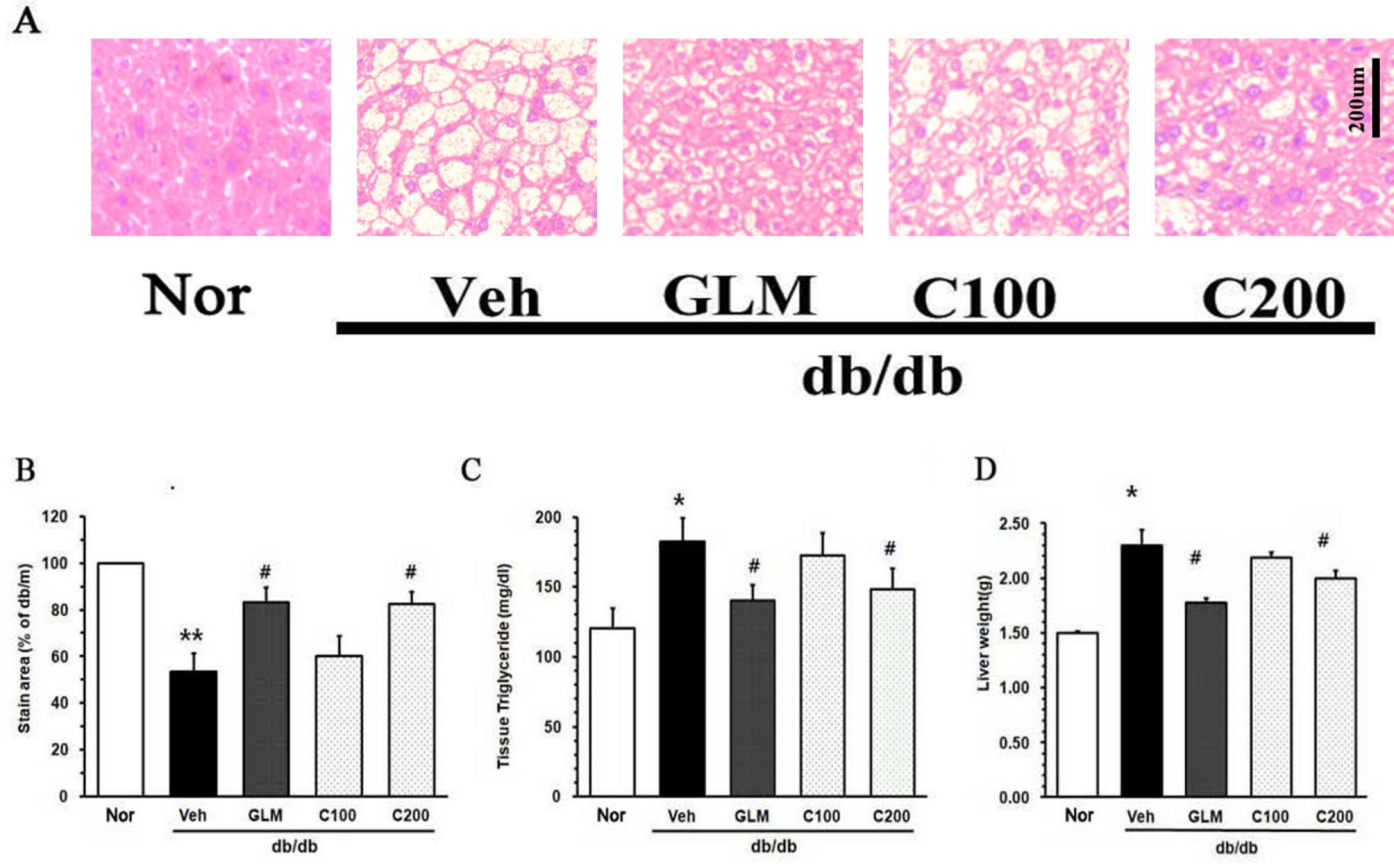
Effects of CJE on hepatic lipid accumulation and histological evaluation in db/db mouse liver. (**A**, **B**) Liver sections were stained with H&E, and graphs show the percentage of the stained area. (**C**) TG in the liver. (**D**) Liver weight. Data are expressed as the mean ± SE (*n* = 6, *p < 0.05 vs. m/m, **p < 0.01 vs. m/m, #*p* < 0.05 vs. Veh)

The results of the histopathologic evaluation of CJE treatment are shown in Fig. 4. H&E-stained sections of the liver in the m/m group showed normal morphology. In contrast, the liver sections from db/db mice showed nuclear displacement caused by lipid droplets and elevated triglyceride levels (Fig. 4A and C). The stained area, which represents the region devoid of lipid droplets, indicates that as the lipid droplets diminish, the level of the graph increases. The Veh group had a smaller stained area compared with the GLM group and the CJE-treated group (Fig. 4B). The lipid droplet and triglyceride levels were decreased in CJE-treated db/db mice in a dose-dependent manner. These results are consistent with the liver weight tread (Fig. 4D). These findings indicated that treatment with CJE reduced liver triglyceride and liver weight in a dose-dependent manner.

### Effect of CJE on the improvement of liver and kidney function

**Fig. 5:**
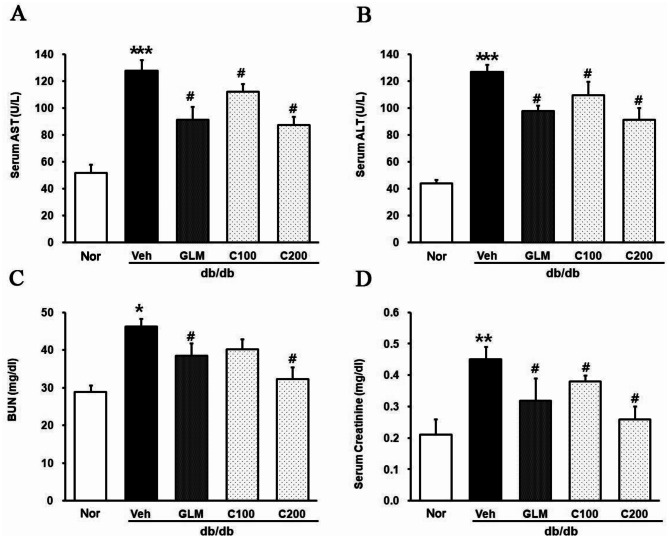
Effects of CJE on the db/db mouse liver and kidney damage. (**A**, **B**) AST and ALT levels reflect liver damage. (**C**, **D**) BUN and creatinine levels reflect kidney damage. Data are expressed as the mean ± SE (*n* = 6, *p < 0.05 vs. m/m, **p < 0.01 vs. m/m, #*p* < 0.05 vs. Veh)

Aspartate aminotransferase (AST) and alanine aminotransferase (ALT) levels reflect the degree of liver damage and creatinine, and blood urea nitrogen (BUN) levels reflect the level of kidney damage. The serum levels of AST (Fig. 5A), ALT (Fig. 5B), creatinine (Fig. 5C) and BUN (Fig. 5D) were significantly increased in the Veh group compared with the m/m group. However, treatement with CJE reduced these levels in a dose-dependent manner. These results suggest that CJE was effective in restoring organ function impaired by insulin resistance.

## Discussion

MetS is characterized by hyperglycemia, hypertension, obesity, and dyslipidemia accompanied by an insulin resistance. MetS with insulin resistance increases the risk of metabolic diseases such as T2DM, CVD, and NAFLD. Insulin resistance is the state of impaired insulin sensitivity and glucose uptake in peripheral tissues such as muscle adipose, and liver tissue [24, 25]. It is major factor in the progression and aggravation of metabolic diseases such as T2DM [26]. Insulin binds to insulin receptors, imitating various signaling pathways that regulate its secretion. An increase in insulin resistance enhances lipolysis, leading to elevated circulating fatty acid levels [27]. This accumulation of fat can cause NAFLD, which in turn contributes to hepatic insulin resistance and can act as a catalyst for several complications Various medications have been developed to treat MetS; however, many of these treatments are associated with various side effects. Consequently, there has been a growing interest in natural substances as alternative therapeutic options. Polyphenols have been demonstrated to influence the pathway by which cells respond to insulin and absorb glucose, thereby preventing oxidative damage [28]. Natural products like CJ contain polyphenols that have positive effects on glucose homeostasis, insulin resistance, and lipid metabolism [16]. Therefore, we studied the effects of CJ on improving MetS including insulin resistance. These findings will help address the gap in research on how CJ affects insulin resistance.

This study demonstrated that CJ reduced body weight, suppressed insulin resistance, and mitigated liver and kidney damage in db/db mice. In obese patients, a 5% reduction in body weight can aid in the treatment of metabolic diseases by decreasing plasma TG levels, improving β-cell function, and alleviating oxidative stress-related MetS [29]. As shown in Fig. 1A and B, diabetic mice treated with CJE showed a decrease in weight gain and inhibition of lipid production in epididymis fat. In patients with T2DM and MetS, key lipoprotein abnormalities such as increased TG and decreased HDL cholesterol levels are observed [30, 31]. These abnormalities include dyslipoproteinemia, such as increased production of VLDL1 and reduced clearance of VLDL, leading to diabetic dyslipidemia and associated complications [32]. As shown in Fig. 3A, TC levels were similar in the GLM, C100, and C200 groups. In Fig. 3B and C, the C200 and GLM groups had similar serum TG and LDL levels. Notably, the C200 group exhibited higher serum HDL concentrations than the GLM group. GLM is a sulfonylurea compound, and sulfonylurea has been widely used as a treatment for type 2 diabetes due to its ability to stimulate insulin secretion from pancreatic β-cells. In this regard, GLM improves lipid metabolism without causing weight gain through mechanisms such as increasing adiponectin levels, thereby preventing complications associated with MetS and diabetes [33, 34]. Due to these effects, GLM is frequently used as a positive control in studies involving db/db mice and metabolic disorders [35, 36]. Our results showed that CJE exhibited effects similar to or better than those of GLM in regard to weight, fat reduction, and cholesterol indicators. As shown in Fig. 4A and B, the Veh group showed a high accumulation of lipids in the liver, with the highest hepatic weight and TG levels. This suggests that CJE was effective in reducing TG and LDL levels and increasing HDL levels.

Abnormalities in lipid metabolism can be caused by insulin resistance and hyperinsulinemia, reflecting the initial stages of a dysfunctional metabolic system. Insulin resistance leads to conditions such as hyperglycemia, hypertension, and fatty liver, which collectively manifest as MetS [37]. As shown in Fig. 2, CJE improved insulin resistance in diabetic mice. The increase in insulin resistance is a mechanism for the development of T2DM and MetS [38]. In our study, we conducted an OGTT and an ITT to measure insulin resistance and comprehensively characterize the metabolic changes associated with insulin resistance (Fig. 2). The OGTT is a method used to measure how the body regulates blood glucose levels after glucose administration in a fasted state, providing insight into glucose homeostasis [39]. Since plasma glucose levels during the OGTT determined by both insulin sensitivity and secretion, it offers a comprehensive estimate of insulin effectiveness [40]. If insulin action is impaired, a wide range of systemic abnormalities, beyond glucose, can be observed, allowing for the prediction of abnormalities in insulin resistance [41]. OGTT measurements demonstrated an improvement in insulin resistance in response to CJE treatment. As shown in Fig. 2A, the CJE group exhibited a glucose gradient similar to that of the GLM group, with even lower blood glucose levels observed at 30 min compared with the GLM group. These results were similarly observed in ITT results. The ITT measures the decrease in plasma blood glucose in blood samples obtained over time from fasted individuals after insulin injection. ITT is effective in predicting the efficacy of insulin sensitizers, and the insulin resistance indicated by ITT results can aid in improving blood glucose control in patients with T2DM [42].

A recent study showed that leptin mediates insulin resistance, a fundamental factor in the development of T2DM and MetS [43]. Leptin, a hormone derived from adipocytes, plays a role in regulating energy balance, metabolism, and the amount of fat stored in the body [44]. Our results showed that CJE treatment led to a decrease in serum insulin and serum leptin levels (Fig. 2D and E). In the C200 group, both serum insulin and leptin levels were significantly reduced compared with those of the Veh group, suggesting that the improvement in insulin resistance by CJE may be from the regulation of energy balance. The HOMA-IR is the most validated method for evaluating insulin resistance [45]. HOMA-IR is used to predict fasting glucose and fasting insulin levels, making it effective for observing various aspects of insulin resistance and pancreatic β-cell function [46, 47]. We observed a significant reduction in HOMA-IR in both the C100 and C200 groups compared with the Veh group (Fig. 2F). The C200 group showed levels similar to those of the GLM group, indicating that CJE may be effective in improving insulin resistance.

Patients with MetS often exhibit other diseases such as NAFLD [48]. Several studies have indicated that obesity can be a risk factor for the development of liver fibrosis, and high HOMA-IR and ALT levels have been reported as predictors of non-alcoholic steatohepatitis (NASH) [49, 50]. The db/db mouse model is characterized by borderline NASH, which results from insulin resistance and hyperglycemia in this model [51]. In our study, the Veh group of the db/db mouse model prominently exhibited hepatic steatosis (Fig. 4A). In addition, we observed an increase intrahepatic TG accumulation (Fig. 4C), a characteristic feature of the db/db mouse, similar to lipid accumulation seen in MetS. In the C200 group, there was a noticeable reduction in the formation of these lipid droplets, and the intrahepatic TG content was significantly reduced compared with levels in the Veh group.

Leptin receptor mutant db/db mice are commonly used as animal models for various MetS, including NAFLD and T2DM. In summary, we found that administering CJE to db/db mice at 200 mg/kg improved fasting blood glucose, serum insulin, serum leptin, and HOMA-IR levels, indicating a potential improvement in insulin resistance. In addition, CJE administration helped reduce hepatic fat accumulation and improved serum lipid metabolism. These finding suggest that further research on the effects of CJE on MetS and related complications could confirm its potential as an effective therapeutic agent.

## Conclusions

This study demonstrated that CJE significantly improved MetS by decreasing body weight, epididymis fat weight, hyperglycemia, dyslipidemia, hyperinsulinemia, and insulin resistance in db/db mice. Additionally, reduced damage to the liver and kidneys was observed in the model mice treated with CJE. Therefore, CJ is very effective in improving MetS and may be a candidate functional food. More research on the CJE through in vitro experiments is required before further development.

## Data Availability

The datasets generated and analyzed during this study are available from the corresponding author upon reasonable request. Any supplementary materials that support the findings of this study can also be made available, provided they adhere to relevant ethical and confidentiality guidelines. Interested parties should contact the corresponding author for further information.

## Abbreviations

AGEs: Advanced Glycation End Products
ALT: Alanina Aminotransferase
AST: Aspartate Aminotransferase
BUN: Blood Urea Nitrogen
CJ: Cirsium japonicum var. Maackii
CJE: Cirsium japonicum var. Maackii extract
CKD: Chronic Kidney Disease
CVD: Cardiovascular Disease
FFA: Free Fatty Acid
GLM: Glimepride
H&E: Hematoxylin and Eosin
HDL: High-Density Liproprotein
HOMA-IF: Homeostasis Model on Insulin
ITT: Insulin Tolerance Test
LDL: Low-Density Lipoprotein
MetS: Metabolic Syndrome
NAFLD: Non-Alcoholic Fatty Liver Disease
NASH: Non-Alcoholic Steatohepatitis
OGTT: Oral Glucose Tolerance Test
PFA: Paraformaldehyde
RAGE: Receptor Advanced Glycation End Products
SGLT2: Sodium Glucose Co-Transporter 2
STZ: Streptozotocin
T2DM: Type 2 Diabetes Mellitus
TC: Total Cholesterol
TG: Triglyceride
VLDL: Very-Low Density Lipoprotein
